# Perspectives of patients, partners, primary and hospital-based health care professionals on living with advanced cancer and systemic treatment

**DOI:** 10.1007/s11764-024-01698-w

**Published:** 2024-10-29

**Authors:** Evie E. M. Kolsteren, Esther Deuning-Smit, Judith B. Prins, Winette T. A. van der Graaf, Linda Kwakkenbos, José A. E. Custers

**Affiliations:** 1 Department of Medical Psychology, Radboud University Medical Center, Nijmegen, The Netherlands; 2 Department of Medical Oncology, Netherlands Cancer Institute, Amsterdam, The Netherlands; 3 Department of Medical Oncology, Erasmus MC Cancer Institute, Erasmus University Medical Center, Rotterdam, The Netherlands; 4 Behavioural Science Institute, Department of Clinical Psychology, Radboud University, Nijmegen, The Netherlands; 5 Radboud University Medical Center, IQ Health, Nijmegen, The Netherlands; 6 Centre for Mindfulness, Department of Psychiatry, Radboud University Medical Center, Nijmegen, The Netherlands

**Keywords:** Advanced cancer, Fear of progression, Illness uncertainty, Interviews, Psycho-oncology

## Abstract

**Purpose:**

An emerging group of patients lives longer with advanced cancer while receiving systemic treatment. This study aimed to investigate psychosocial aspects of living longer with advanced cancer, and experiences with psychosocial care, from the perspectives of patients, partners, and health care professionals (HCPs).

**Methods:**

From May to December 2020, participants were purposively selected. In-depth, semi-structured interviews were conducted by video or phone call, containing open questions regarding psychosocial aspects and psychosocial care in oncology. The data was analysed following thematic analysis, leading to overarching psychosocial themes and indications for optimal organisation of psycho-oncological care.

**Results:**

Fifteen patients, seven partners and eleven HCPs were interviewed. The main psychosocial aspects were increasing loss in several life domains, complexity of making life choices, ongoing uncertainty, and fluctuating fear and hope. Partners were affected by their loved ones’ condition and reported to put themselves second for longer periods of time, while sometimes missing adequate support. HCPs were challenged by addressing the altering psychosocial needs of patients, and tools to identify those in need for psychosocial support are currently lacking.

**Conclusions:**

Living longer with advanced cancer presents unique challenges for patients and their partners, as well as for HCPs in delivering optimal psychosocial care.

**Implications for Cancer Survivors:**

Identifying and addressing patients’ psychosocial needs from an early stage on, appointing a central hospital-based contact person, limiting the waiting time between scans and consultations, and addressing the partners’ wellbeing are suggestions to organise optimal psychosocial support in advanced cancer.

**Supplementary Information:**

The online version contains supplementary material available at 10.1007/s11764-024-01698-w.

## Introduction

The introduction of novel systemic treatments and treatment combinations, including the development of immunotherapy, targeted therapies, and more recently antibody–drug conjugates, has led to improved progression-free and overall survival in several types of cancer [1–4]. As a result, an increasing group of patients lives longer with advanced cancer [5–10]. In this study, ‘patients with advanced cancer’ refers to patients diagnosed with metastatic, or locally unresectable, incurable cancers with a poor and uncertain prognosis, receiving systemic treatment aimed at prolonging life. Disease courses of advanced cancer are diverse, and an individual patient’s life expectancy is complex to predict given the uncertainty of how patients respond to newer treatments [11].

Although it is encouraging that people responding to treatment live longer with a terminal illness that would previously have advanced quickly, living longer with advanced cancer is associated with psychosocial burden. Individuals live with the certainty of cancer progression at an uncertain moment in time [12–16]. The patient’s awareness of their incurable cancer progressing, leading to a forthcoming death, might differ from the psychosocial experiences of cancer patients or cancer survivors treated with curative intent [12, 13, 17, 18]. Patients with advanced cancer continuously shift between hope and despair, alternating between living with and dying from their disease [16, 19, 20], and fears regarding progression and death are commonly reported issues [12, 19, 21, 22]. To deal with the psychological burden of their condition, close relations and social support are reported to be of great importance for cancer patients [19, 21, 23]. However, patients living longer with advanced cancer also experience the misunderstanding of their condition by others [16, 21, 23]. Partners of patients are an important source of support. In addition to their role as informal caregiver, they must deal with the consequences of living with a partner with advanced cancer, which negatively influences their own future perspective and wellbeing [23]. Both patients and their informal caregivers, including partners, reported unmet needs in their psychosocial wellbeing and received support throughout a long-term advanced disease trajectory [24]. Nevertheless, there is limited research on the specific psychosocial impact experienced by partners of patients with advanced cancer responding long-term to systemic treatment [25].

With an increasing number of patients living longer with advanced disease, the various involved health care professionals (HCPs) are challenged to adapt their clinical practice routines to guiding patients through a longer, but uncertain disease trajectory [26]. Although there is increasing literature on the psychosocial aspects faced by patients living longer with advanced cancer, there is a lack of knowledge on HCPs’ experiences in guiding this group of patients and what is needed to optimally guide those patients and their loved ones through a longer-term, uncertain, disease trajectory [16, 27]. It is therefore important to further explore these psychosocial aspects from several perspectives and to explore how they evolve during a prolonged disease trajectory. Increased knowledge on the psychosocial experiences of both patients and their partners will support HCPs to provide better psychosocial guidance and to address those psychosocial issues relevant at different stages of a prolonged disease trajectory. The aim of this interview study was to explore the psychosocial aspects of living longer with advanced cancer and systemic treatment and how these aspects evolve through time, from the perspectives of patients, partners and HCPs, together with experiences on psychosocial care in oncology. Increased knowledge can increase awareness for the unique struggles faced by patients and their partners and helps to inform HCPs on how to organise optimal psychosocial support.

## Methods

In-depth semi-structured interviews were conducted with patients, their partners, and HCPs. Results are reported per the ‘Consolidation Criteria for Reporting Qualitative Studies’ (COREQ) guidelines (Supplementary File I) [28].

### Participants and recruitment

#### Patients

From November 2019 to December 2020, adult patients diagnosed with a form of advanced cancer who were receiving systemic treatment were purposively recruited via their HCP at the departments of Medical Psychology, Medical Oncology, Lung Disease, or Urology of the Radboudumc hospital in Nijmegen, the Netherlands, to offer a range of perspectives based on age, sex, type of advanced cancer and type of systemic treatment. Two patients self-referred for participation by contacting their HCP after reading about the overall research project of which the interview study is part, in an oncology magazine. Patients were eligible to be interviewed if they were (1) physically well enough to participate in a one-hour-interview, (2) had sufficient command of the Dutch language, and (3) were able to give informed consent. Patients were first informed about the study by their treating HCP (oncologist, clinical nurse specialist (CNS) or psychologist) during a regular consultation, who provided them with a detailed information letter including an informed consent form. Patients who received the information letter were contacted by phone by the researcher (EK) within two weeks. The researcher provided additional information and answered any questions patients had regarding the study. Participants returned their signed informed consent form via post or email to the researcher before participating in the interview study.

#### Partners

Partners of participating patients were invited to take part, and the same three eligibility criteria as stated before were applied. An informed consent form was signed and returned by email or post by partners before enrolment.

#### Health care professionals

HCPs working in the hospital or primary care with a professional background in treating patients with advanced cancer receiving systemic treatment, and sufficient command of the Dutch language, were purposively sampled via the network of the researchers in this study. Participants included medical specialists, clinical nurse specialists, psychologists, social workers, and general practitioners. HCPs received an information letter directly from the researcher and signed informed consent before inclusion.

### Semi-structured interviews

Due to COVID-19 restrictions at the start of the study, the interviews were conducted through video call or phone call, based on participant preferences, at a convenient time for the participant. The interviews were performed by a female PhD candidate with training in conducting qualitative research (EK), and a few interviews were attended by the (female) principal investigator (JC). Three separate semi-structured interview guides were developed: one for interviews with patients solely, one for patient and partner jointly, and one for the HCPs (Supplementary File II-IV). The interview topics were derived from literature on psychosocial outcomes in advanced cancer, including preliminary results from a scoping review that was conducted concurrently by the research team [21]. Additionally, EK attended consultations of patients and their clinical psychologist (JP) and spoke to three patients individually. Next, the interview guides were developed by EK which were discussed and adjusted by members of the research team (JC and JP). The interviews consisted of open questions exploring the following topics for patients: (1) psychological aspects of living longer with advanced cancer and systemic treatment (including thoughts, emotions, triggers, behaviour, coping); (2) social aspects of living longer with advanced cancer and systemic treatment (including the impact on social relationships and perceived social support); and (3) experiences with psychosocial care. The interviewer began with an introductory question about diagnosis and treatment history to help acclimate the patient and to get a general impression of the disease trajectory. Interviews with both patient and partner started jointly with the same introduction question about diagnosis and treatment history, whereafter psychological and social aspects were explored. Both the patient and partner were individually addressed on these topics. HCPs were interviewed about (1) their professional background in working with people with advanced cancer and systemic treatment, (2) their view on the psychosocial aspects of living with advanced cancer and systemic treatment, (3) their view on the organisation of psychosocial care for patients with advanced cancer, and (4) their personal experiences of treating those patients. All interviews were audio recorded. Collection of data continued until saturation. Saturation deemed to be reached when the pre-defined desired variation in patient characteristics and professional backgrounds was met, and no new (sub)themes were identified through data coding. The researchers regularly evaluated whether new themes came up, and topics were elaborated on in subsequent interviews if needed.

### Qualitative data analysis

**Fig. 1:**
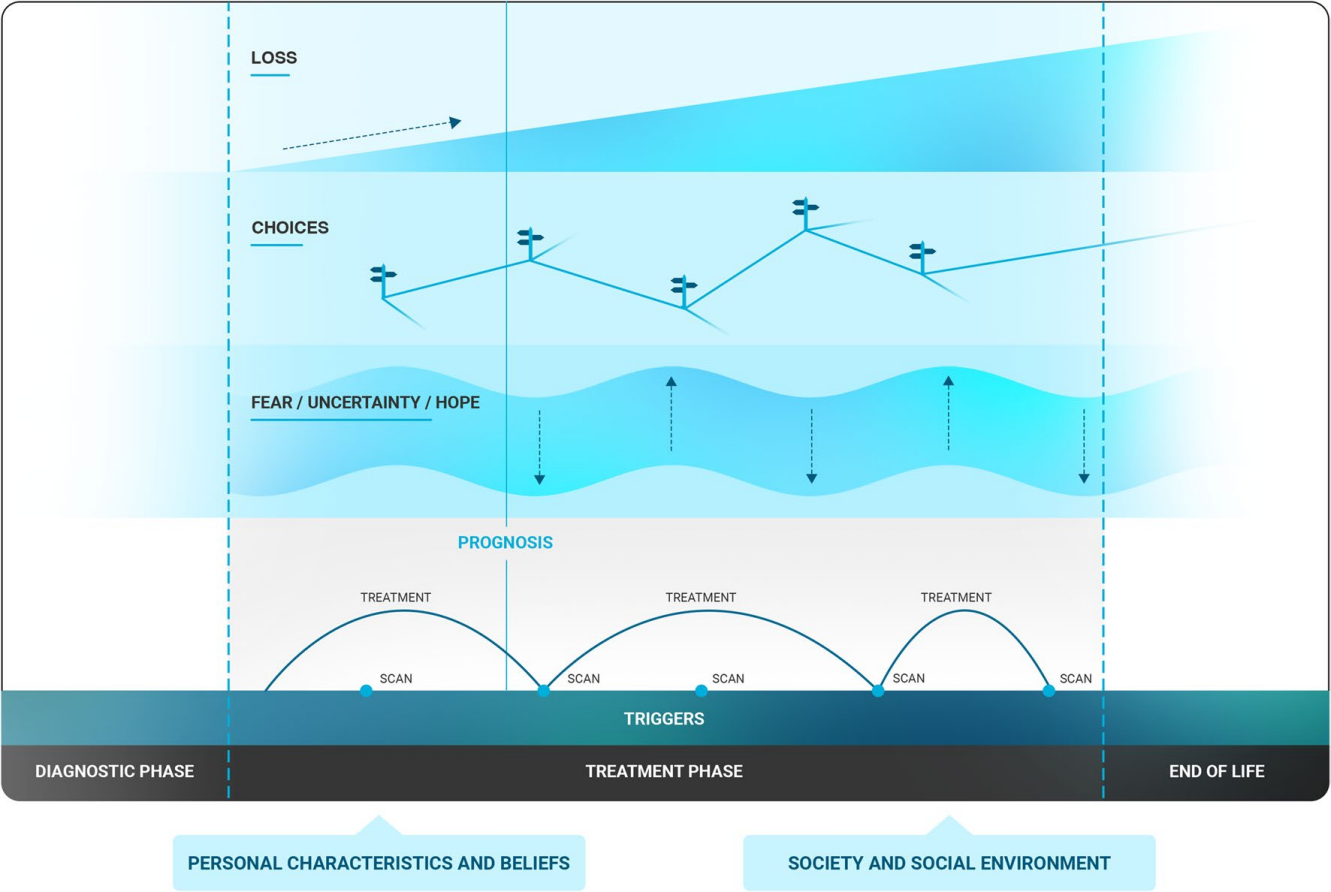
Psychosocial aspects of living with advanced cancer and systemic treatment

Sociodemographic and clinical characteristics of patients were obtained via patients themselves and their treating HCPs, and participating HCPs provided details on their age and profession. Interviews were transcribed verbatim and transcripts were coded and analysed using thematic analysis [29]. The software program Atlas.ti version 22 was used to manage the data. Initial codes were generated directly from the data and applied by two researchers independently (EK and ED) following the principles of open coding. After both researchers independently coded one interview, the applied codes and code names were discussed to reach consensus, and if needed, the principal investigator (JC) was consulted to discuss discrepancies. The initial coding of the interviews took place simultaneously with conducting the interviews, and if needed based on the coding results, interview guides were adjusted. After ten patient-partner interviews and eight HCP interviews, the remaining eight interviews were coded by EK and reviewed by ED. Disagreements were discussed and again the principal investigator (JC) was consulted if needed to jointly solve discrepancies. The initial codes were merged, reformulated, and/or summarised by EK and ED into thematic code groups reflecting topics related to the research question. Thematic code groups were further clustered and summarised into broader (sub)themes. This process involved going back and forth between the data, codes, code groups and themes, which were regularly discussed and revised by EK and ED. The final code book was drawn by EK and presented to the research team, which included the principal investigator JC, clinical psychologist JP, oncologist WG, and researchers LK and ED. Following comprehensive discussions about the evolvement of the psychosocial aspects through time with the research team (JP, WG, LK and JC), EK and ED created an illustrative figure visualising the main psychosocial outcomes (Fig. 1), which was further discussed and refined by the research team (Fig. 1).

## Results

### Participants

Between May and December 2020, 26 interviews were conducted (with 15 patients, in 7 cases including their partner, and 11 HCPs) (Tables 1 and 2). Seventeen patients were approached, of whom one patient decided not to participate after reading the information letter, and another patient could not participate because of illness. Eight male (53%) and seven female patients (47%) with a mean age of 59 (10.7) years were interviewed. They had varying advanced cancer diagnoses, including melanoma, gastrointestinal tract cancer, lung cancer, breast cancer, gastrointestinal stromal tumour, leiomyosarcoma and multiple myeloma. Seven partners (four women and three men) with a mean age of 63 (17.8) years participated. All seven couples were married. Nine interviews (60%) took place via video call and six interviews (40%) via phone call, with an average duration of 87 min (range 58 min to 2 h and 45 min). In two patient-partner interviews, patient and partner were interviewed separately. In the other five patient-partner interviews, patient and partner were both present, and parts of the interview were directed to each individually, as described in the “Methods” section.

**Table 1:** Patient and partner characteristics

	Patients	Partners
Number of participants	15	7
Mean age (SD)	59 (10.7)^a^	63 (17.8)^a^
Sex, *n* (%)		
Female	7 (46.7)	4 (57.1)
Male	8 (53.3)	3 (42.9)
Relationship status, *n* (%)		
Single	2 (13.3)	0 (0)
Married	13 (86.7)	7 (100)
Metastatic cancer diagnosis, *n* (%)		
Melanoma	5 (33.3)	N/A
Gastrointestinal tract (*liver, stomach, colon*)	3 (20.0)	
Lung	2 (13.3)	
Breast	2 (13.3)	
Gastrointestinal stromal tumour	1 (6.7)	
Leiomyosarcoma	1 (6.7)	
Multiple myeloma	1 (6.7)	

*N/A* not applicable

^a^Missing data of 1 participant

**Table 2:** Health care professional characteristics

	Health care professionals
Number of participants	11
Mean age (SD)	45 (5.3)
Sex, *n* (%)	
Female	7 (63.6)
Male	4 (36.4)
Profession, *n* (%)	
Clinical nurse specialist	2
General practitioner	2
Medical oncologist	2
Pulmonologist	1
Urologist	1
Registered clinical psychologist	1
Registered health care psychologist	1
Social worker	1

Eleven HCPs were invited, and all accepted their invitation to be interviewed: four medical specialists (two medical oncologists, one pulmonologist and one urologist), two CNSs, one social worker and one registered clinical psychologist, all from two different hospitals, one registered health care psychologist from a mental health care institute for psycho-oncology, and two GPs working in the surroundings of Nijmegen participated, with a mean age of 45 years (5.3) and 64% being female (Table 2). All interviews took place through video calls with an average duration of 51 min (range 41 to 59 min).

### Psychosocial aspects of living with advanced cancer and systemic treatment

The psychosocial aspects of living longer with advanced cancer while receiving systemic treatment, and experiences with psychosocial care were explored in the interviews. Figure 1 describes the erratic interplay of the most prominent, overarching psychosocial themes of the advanced cancer trajectory characterised by a long-term treatment phase, recurrent scans, and living towards or surviving the prognosis, including (1) increasing loss in multiple life domains, (2) the complexity of making life choices, and (3) ongoing uncertainty and fluctuating fear and hope. The perspectives of patients, partners and HCPs on these topics are summarised in main themes below.

#### Increasing loss

Patients and partners described losses across multiple life domains. A paramount loss for patients was the anticipated normal course of healthy aging, and patients had to accept the loss of their ‘taken for granted’ physical health and future perspective. A sudden confrontation with severe illness and an approaching death often heightened patients’ awareness of experiencing significant seasonal moments, such as their birthday or Christmas, for potentially the last time. They became aware of future events they might not be able to witness anymore, and consequently, special dates or events became more heavy-weighted for some when time passed. Patients experienced fluctuating feelings of anger, fear, depression, sadness and hope, influenced by alterations in their health and treatment outcomes, such as a bad scan result, which constantly invoked their resilience.

Patients experienced substantial losses regarding their working life. All were unable to continue their work as they used to before their diagnosis. They felt guilty about their unavoidable absence at work, and the loss of their ability to contribute to society, and sense of value was difficult for patients. Most patients struggled with the sudden changes in their daily (working) life after their diagnosis, although patients close to retiring sometimes felt relief upon deciding to quit their job earlier. Patients expressed the sense of losing (part of) their personal identity with losing their job, and for some, this had an impact on their social relationships as well. Patients felt not able to come along with their peers, creating distance towards others. Younger patients were especially confronted with their losses, when comparing themselves with others’ job opportunities and life perspectives. Most patients therefore preferred to retain their job in some way if possible, for example with reduced hours, and tried to stay in contact with colleagues otherwise.

*‘You see, your job, and especially in my profession [medical specialist], it is part of your identity, and you don’t get there easily. […] And from one day to another, you lose it all. And I struggle a lot with that. And that might be the reason I don’t like to go to parties and those kinds of things anymore. Everyone in our group of friends is doing good. Everyone has a good salary, and they talk a lot about their jobs. And in fact, I am the outsider. They don’t ask about you anymore, so you feel like you are retired, but you didn’t fulfil a working life’.* (Male, 54, multiple myeloma)

Partners’ future perspectives changed directly with their spouse being diagnosed with advanced cancer. However, ‘normal life’ proceeded for them, challenging them to balance their professional obligations with their role as informal caregiver at home. Partners adapted their working hours, resulting in reduced working hours to keep up with their caring tasks at home, or increased working hours to prevent financial stress because of the job loss or reduced income of their partner. Partners did not structurally feel supported by their employer in their needs for adjusted working hours for uncertain periods of time or for example with granting permission for a long-term leave. These struggles on top of the emotional burden of having a partner with an advanced cancer diagnosis made partners feel stressed, anxious, and sometimes even burned-out.

#### Complexity of making life choices

Patients were confronted with the complexity of making life choices because of their poor but uncertain prognosis. For example, together with their partners and their specialists, patients actively made medical decisions regarding continuing or discontinuing treatment and the planning of scans, influencing their future perspective. They balanced continuing treatment and the possibility of prolonging life, with the (potential) treatment side effects and the negative psychological impact of living longer in uncertainty. These decisions affected patients’ physical and psychological wellbeing and the future perspectives of them and their loved ones as well. Patients and their partners felt sad, mad, or depressed regarding the diagnosis from time to time, but were often capable of deemphasising their negative thoughts and staying positive. Younger patients had motivations that differed from older patients to base their choices on. For example, patients with (young) children had a stronger motivation to continue medical treatment to live longer, while older patients valued quality over quantity of life. However, all participants desired to spend their remaining time in a meaningful way and had to decide on how to do so. Patients exemplified to focus on for them personal meaningful things in life, such as the ability to do sports, to perform household tasks independently, to go on holidays, or to spend time with family and friends. Their physical condition made them adjust their goals and live day by day. Making plans and decisions for the future was therefore sometimes problematic and in particular stressful for those patients who felt the inevitable end of life approaching with time passing. This concerned relatively marginal decisions, such as the prolonging of a sports subscription for another year, or buying concert tickets in some time from now, as well as bigger decisions, concerning family planning for younger couples, financial issues, arranging a life insurance or moving to another house for their partner to live in after passing away. When time passed and patients had generally good treatment results, they and their partners dared to ‘grant’ themselves more future time, which made it easier to make plans or decisions further ahead. Patients who had anticipated an early death sometimes looked back with remorse of having done things differently if they would have known to live longer. For instance, a patient with good scan results for years now regretted the irreversible decision of permanent work disability, leading to increased feelings of sadness and depression. Another patient looked back on forgone future plans, which had affected his partners’ life as well:

*‘What we [patient and partner] thought after retiring was buying a small house with a big garden close to the forest. That didn’t happen. It didn’t seem to be meaningful anymore’. Interviewer: ‘and that was ten years ago’. Participant: ‘Yes, that’s right’.* (Male, 73, metastatic leiomyosarcoma)

#### Ongoing uncertainty, fear and hope

In describing their emotions, participants often mentioned uncertainty, fear and hope. They experienced an ongoing uncertainty regarding various aspects of their life, including their treatment response, cancer progression, and whether effective treatment options would be available when needed. Ultimately, they were aware of the uncertain future perspective they had and the complexity of continuing their life. This continuous living with uncertainty went along with feelings of fear and hope, fluctuating through time. Which emotions predominated depended for example on recent test results or their physical condition. Patients who had not initially noticed the onset or progression of their cancer interpreted bodily sensations such as pain or cough as possible indications of the cancer progressing, leading to increased feelings of fear and uncertainty. Most patients were aware of their initial prognosis and they lived up to it. Surviving their prognosis was perceived by many as an important mile stone, but led to increased uncertainty and fear when making future plans beyond that prognosis as well. In case of (recurrent) good scan results, surviving the prognosis increased hope to live longer, fostering the belief to be able to ‘beat the statistics’. Conversely, others anticipated that surviving their prognosis meant that it could not take much longer before progression would occur, increasing fear of progression and decreasing hope. Although patients gradually became more adept over time at settling down with their disease, the continuous adapting was perceived challenging.

*‘People really have to adjust during their treatment phase. In the beginning they just hope that treatment works, but then they have to adapt to the fact that treatment is effective, and they have to continue living. And that is very difficult’.* (CNS, Male, 45)

Patients participating in phase 1 clinical trials mentioned that the uncertainty regarding the unknown treatment effect of their novel trial medication was particularly difficult to deal with. They perceived their experimental treatment as their last option to prolong life, which made them feel restless, but it gave room for hope as well, translated into the hope to obtain a normal life expectancy with consecutive new treatments available, and even the hope to be cured at last.

Consultations about scan or blood results were crucial moments for both patients and their partners, characterised by heightened levels of stress and fear, typically starting in the week(s) leading up to the scan, persistent until the consultation. Patients frequently found themselves unable to look beyond the next scan appointment, limiting their ability to see a longer-term future for themselves. Patients noted the emotional burden of the long waiting time between undergoing a scan and the consultation with the oncologist about the results, which often took up to a week. Some patients could view the results in their electronic patient record beforehand, which led to increased or decreased levels of stress, depending on their interpretation of the results.

*‘On the day itself, it’s often relatively okay. Until I’m there for the appointment. When I am about to get the results, and I enter that waiting room, that’s when it starts. You hear approaching footsteps, and they already call someone in, that’s when it takes on a life of its own. It’s just the fear. So, when I enter, I prefer them just saying it right away, without first asking “how are you doing?”’* (Female, 62, metastatic liver cancer)

Patients and partners dealt in various ways with the recurrent cycle of emotions around scans. For example, by living in the moment and trying to keep themselves distracted. With time, fear and anxiety around scans increased for some, and decreased for others, sometimes influenced by good or bad test results from the past. Partners accompanied their loved ones with consultations and shared in emotions with good or bad results. However, anticipating the results was complex for them, as they had to rely on the patients’ expectations of the outcomes. To deal with the emotional burden of recurrent scans, patients sometimes preferred to alter the frequency of scans and discussed this with their specialists. This could lead to discrepancies in desired scan frequency between patients and their partners.

#### Impact on social relationships

Supportive social relationships and the recognition of others was essential to patients. However, both patients and partners at times felt misunderstood by their social environment, as others failed to grasp the extent of the patient’s disease. Without noticeable physical impairments or changes in appearance especially, patients were leading seemingly normal lives for the outside world, sometimes triggering inappropriate responses. This misunderstanding led to feelings of anguish and even the loss of friendships.

*‘But on the outside, you don’t see it. Then people say “when are you going on vacation again?”, meaning if you can go on vacation, you can also go to work, because they don’t see your illness. At first, I took it personally. They said it with a half-smile, but now and then you could detect an undertone of “hey, he can go on vacation and he’s not working”’.* (Male, 58, metastatic lung cancer)

Patients struggled with the fact that their condition negatively affected their close ones. They found it depressing to realise the emotional burden of their illness and the prospect of an early death for their loved ones, and the impact their condition had on the lives and future perspectives of close ones. In particular for those they were living with, including children and partners.

*‘I have also changed a lot. I am often miserable. I think I also dwell much more on what is coming, and on the one hand, you know, I want everything to go on, but it doesn’t. A lot has changed for them [partner and child] too. Yes, I think my partner feels like he’s standing still in life sometimes because I can’t go on long vacations or do certain things anymore, or because I’m tired in the evenings. Yes, that’s hard. That’s very hard’.* (Female, 51, metastatic breast cancer)

#### Unique challenges within the patient-partner relation

Patients emphasised the importance of a strong and supportive relationship with their partner in dealing with their condition. However, not all patients and partners were able to communicate about their emotions with each other. In some cases, patients and partners tried to stay emotionally strong and optimistic to shield each other from their emotional burden, while in other cases patients and partners had conflicting perceptions of the patient’s diagnosis and consequences, leading to differing, misunderstood emotions and sometimes conflict.

As the patient’s informal caregiver, partners self-sacrificed for long but uncertain periods of time. Their own future plans and sometimes psychosocial wellbeing were strongly affected. Often, patients were aware of their partners putting their own needs and future plans on hold, but not always able to support them. Partners were preoccupied with their partner’s disease and prospect of an early death and had fears regarding the last part of their partners’ life, a future life without their partner, financial issues, or not being able to reach life goals. Strong feelings of fear were for example triggered during close moments together or by the occurrence of cancer in their social network or in the media. For some partners, fears increased with time, while others found themselves less preoccupied on most days, gradually adapting to the situation. In some cases, with recurrent good results, partners were able to return to normal life as much as possible, allowing the disease to fade into the background, sometimes in discrepancy with the patients’ experience.

*‘There are many aspects to the entire partnership. On one hand, you’re the one who, in my case, has to take care of him because he couldn't do anything, while emotionally you’re in a sort of uncertainty bubble because I’ll be left behind without him. […] As a partner, I think you have even more things to deal with at such a moment than the patient themselves’.* (Partner, female, 34)

Patients living alone faced unique difficulties. In the course around scans especially, single patients expressed to feel lonely and they missed the ability to share their emotions and worries with someone in their everyday life. They struggled with having to actively ask others to comfort them with their anxiety and stress at certain moments, which made them feel vulnerable and helpless. Patients struggled with starting new relationships as well. One single patient told to have a desire to find a partner, but to be reluctant with finding someone because of the uncertainty of her future perspective.

*‘I would actually quite like to find a new partner. But I’m incredibly hesitant about it. […] What do I have to offer? I mean, someone might like me, but they also get my uncertain future for free. And someone has to be willing to accept that. And when do you bring it up? I can’t just put on my Tinder profile, “Hi, I have cancer, who likes me?”’.* (Female, 67, metastatic gastric cancer)

#### Personal characteristics and disease perceptions

In describing their emotions, participants referred to their personal characteristics. They described themselves as a positivist, as being resilient, or as a worrier. Patients perceived to have changed as a person because of their illness, for example in becoming emotionally unstable, becoming better at putting themselves first, at living in the present, or at appreciating the small things in life. The disease perceptions they had were diverse, for example ranging from the belief that recurrent good scan results indicated the cancer curing, to the belief that they only had little spare time left in good condition. Patients trusted that treatment side effects indicated a treatment effect. They felt ‘fortunate’ with treatment success or ‘unfortunate’ with cancer progression and running out of treatment options. Some believed that a healthy lifestyle added to their treatment success, while others searched for a cause of their disease in themselves (e.g., stress as a cause). Most partners had faith that the patient still had years to live. HCPs noted that terms like ‘palliative’ and ‘metastatic’ were profoundly terrifying for patients and partners, because of the connotation of a poor prognosis and imminent death.

### Complexity of providing optimal psychosocial support

Although patients experienced a variety of sometimes long-term psychosocial struggles, they perceived this to be normal given their condition, and not all of them were in need for (additional) psychological help. However, when required, patients did not always receive the desired psychosocial help due to a lack of knowledge on where to find support, incorrect or no referral, or long waiting lists. They expressed their desire for a standard offer from their specialist on where to find psychosocial guidance or information outside the consultation room, for whenever psychosocial support would become needed.

#### Long-term guidance of patients

With patients living longer with advanced cancer, specialists faced challenges in providing comprehensive long-term care. With the expansion of new treatment options and personalised medicine in advanced cancer, oncologists needed increased time to discuss comprehensive medical information with their patients. As a consequence, less time was available to address psychological wellbeing, although the specialists acknowledged the unique psychosocial struggles of living with advanced cancer, and the importance of addressing psychosocial wellbeing in the consultation room. In addition to lack of time, some oncologists mentioned the lack of competences to properly address psychosocial wellbeing and provide psychosocial support, and mentioned a lack of knowledge and information where to refer patients to. Additionally, patients were sometimes reluctant in being open about the emotional burden of their disease as they feared not being considered eligible by their specialist to receive sequent or new treatment options. Partners were hesitant about when or where to seek psychosocial help for themselves if needed, because (medical) guidance mainly focused on the patient.

*‘With increasing medical opportunities, there is less room and time for psychosocial guidance. […]. When I started as a doctor, lung cancer meant a very poor prognosis. You could try chemotherapy to prolong life with a few months, but that was it. It was bad news; however, it was clear, and you had a lot of time for advanced care planning. I find that very challenging nowadays’.* (Oncologist, female, 40)

Similar to oncologists, psychologists and social workers are confronted with the guidance of an emerging group of patients and their partners for extended periods of time, and consequently build up a close relationship. Patients presented themselves with complex, recurrent, existential issues on how to live their remaining life. For psycho-oncological professionals, it remained a challenge how to optimally provide the requested long-term support. Besides offering psychological treatments to cope with for example specific fears, they sometimes provided ongoing guidance, or guidance intensified during pressing phases as preferred by the patient, such as around a scan, with changes in treatment, or personal changes or complex life choices. Psychologists attempted to empower patients to cope independently as an alternative to continuing psychological consultations, for example with the help of their own social network, and if needed by involving another HCP such as their own GP, but organising optimal support remained challenging.

*‘In general, it is a much longer process, including very long pauses sometimes. You may not see someone for a while, but then something happens, changes in their personal situation, or treatment, which makes it difficult for them to cope, so they come back for a while. Sometimes it’s just to check in and make sure everything goes well again. And, I think that generally, it’s more about guidance and helping someone get clarity on their situation and what they can do, rather than real psychological treatments. It’s not entirely separate, though, it overlaps; there is certainly a therapeutic aspect in those conversations, but sometimes it’s more about guidance than actual treatment’. (Clinical psychologist, female, 50)*

#### Addressing end-of-life aspects directly after diagnosis

Patients with a close relationship with their GP regarded their GP as more low-key and better accessible to reach out to in case of issues than their hospital-based specialist. This included medical as well as psychosocial aspects. GPs preferred to remain involved with the patient and their close ones throughout the entire disease trajectory. They therefore regularly contacted their patient to ascertain whether support was demanded or not. GPs felt the necessity to discuss aspects of the end-of-life phase with patients from an early phase on, preferably directly after the patient was diagnosed. This included discussing possibilities and preferences regarding dying and death in the patients’ home situation, such as euthanasia. Yet, if patients were in an active treatment phase aimed at prolonging life, discussions about the end of life were hampered. In addition, GPs were not consistently engaged and up to date about the patients’ medical condition, which could complicate providing optimal support. They stressed the importance of being informed by other involved (hospital-based) HCPs and called for improved awareness of responsibilities regarding the patient’s wellbeing and improved communication among involved HCPs in primary care and the hospital.

*‘But if you’ve just heard in the hospital that, if you’re lucky, you might still have another five years ahead, at least with immunotherapy, then as a patient, you get a different perspective. And you’re less open to those conversations about end-of-life’.* (GP, male, 55)

#### Central role in psychosocial guidance

The CNSs participating in this interview study both had a central role in (psychosocially) guiding patients during their disease trajectory. However, the role of CNSs differs among hospitals. They reported to have more time to engage with their patients and loved ones compared to oncologists, allowing for greater emphasis on patients’ psychosocial wellbeing during consultations. They valued to follow up patients for longer periods of time and appreciated the strong connections established with patients and partners because of this longer-term involvement. Patients and partners welcomed a hospital-based caregiver functioning as fixed contact to reach out to in case of physical or psychosocial issues, with whom they build up a strong bond since their diagnosis. Other HCPs mentioned the added value of a central hospital-based contact person for patients, filled in by for example a CNS with oncological and psycho-oncological expertise, and stressed the importance of a focus on psychosocial wellbeing for patients and partners.

*‘Case managers are very helpful, because you can immediately reach out to them, you can share your story and they respond immediately to questions’.* (Partner, male, 68)

## Discussion

This interview study demonstrated that increasing loss in various life domains, making complex life choices, ongoing uncertainty, and fluctuating fear and hope around (recurrent) medical events such as scans or changes in health status or medical treatment were significant psychosocial aspects for patients living longer with advanced cancer and systemic treatment (Fig. 1), which is in line with previous literature [8, 15–17, 19, 24]. Our interview study highlighted the partners’ perspectives and the challenges of HCPs in providing long-term psychosocial care, thereby giving insight in how to optimise psychosocial support for both patients and their partners during a prolonged advanced cancer trajectory.

One of the psychosocial issues that highlighted the different perspectives of patients, partners and HCPs centred around fear, especially in the timeframe around scans, referred to as ‘scanxiety’ [30–33]. Patients received recurrent scans (e.g. every three months) to closely monitor disease progression. They reported to live from scan to scan and especially the waiting time between undergoing the scan and the consultation with the oncologist, which could last up to one week, was perceived as nerve-racking [31]. For those patients who have access to Electronic Health Systems, the option to read the scan result before consulting the specialist is increasingly available. However, scan or test results are difficult to interpret without medical expertise or a layman’s version and could leave patients in doubt or with an inappropriate perception about their results. To decrease scanxiety, waiting times could be minimised as much as possible. However, this is challenging and puts extra pressure on the radiologist and oncologist to interpret and share the results instantly [33].

Another challenge for specialists is to discuss comprehensive medical information with the patient in the limited time available per consultation. Besides the patients’ physical condition, addressing and monitoring the patients’ psychosocial wellbeing is needed in the consultation room and calls for a different approach compared to curatively treated patients, as other psychosocial aspects are prominent, including ongoing uncertainty, fear of progression, and questions regarding how to continue life with advanced disease. Managing the uncertainty and enhancing a sense of control can be supported by HCPs through providing clear and concrete information regarding prognosis, treatment options, and expected waiting times for scan results. Some patients may benefit from counselling throughout their illness trajectory, aimed at helping them accept the uncontrollable aspects of their condition and to regain a sense of control over daily life. Because it is complex to predict how fast the patient’s physical health will deteriorate, communication about life expectancy and end of life is equally important, but complicated [11, 27, 28]. However, it is beneficial for both HCPs and patients to discuss the patients’ needs, goals and preferences regarding treatment, psychosocial care, and how to spend their lives, from an early stage on [34]. So-called advance care planning (ACP) is meant to define and set patients’ needs, goals and preferences, to discuss these with their loved ones and HCPs involved, and to record, and if needed, review these goals and preferences, aiming at arranging better fitted care and better quality of life for patients and close ones near the end of life [35, 36]. However, patients receiving treatment to prolong life might not be open to discuss end-of life issues. Nonetheless, it remains uncertain whether patients receiving systemic treatment will face rapid decline or live longer with advanced cancer, and immediate discussion of end-of-life preferences is of great importance and contributes to overall quality of life. In line with the aims of ACP, HCPs mentioned in the interviews that patients’ preferences are inventoried preferably directly after patients receive their advanced cancer diagnosis and further throughout the disease trajectory, since preferences might change and disease trajectories are uncertain. It is of great importance to engage all involved HCPs in ACP, in both primary care and the hospital, and to enhance mutual communication.

In addition to discussing the patients’ psychosocial wellbeing, awareness for the partners’ wellbeing is essential, as the results of this study showed that partners also experience uncertainty, fear, hope, and an impact on their social, work, and future life. Partners often provide long-term care to patients at home for an uncertain period of time. Additionally, they live with the emotional burden of their partner being incurably ill and a future perspective of losing their spouse. By providing long-term care, partners contribute to patients’ quality of life and potentially reduce hospitalisations [37]. With advanced cancer, long-term care provided by partners is without a clear perspective of how long this will be needed, but with the certain prospect of death of their spouse. These circumstances are utmost challenging and self-sacrifice and caregiver burden are common [38]. To support partners, it is essential to increase awareness for the wellbeing of partners in the consultation room. They should be involved in discussions about psychosocial wellbeing and goals and preferences regarding treatment, care, and end-of-life [39]. Patients and partners may differ in their psychosocial experiences of living with advanced cancer and the preferences they have regarding treatment, care, and how to continue life. A recent survey study among caregivers of advanced cancer patients reported that about 25% of patient-partner couples does not have the same prognostic information preferences regarding mortality risk, complicating discussions in the consultation room [40]. Additionally, a considerable proportion of the couples has discrepant perceptions of the actual prognosis [40]. For HCPs, these discrepancies may not be obvious, and it is challenging to assess the capability of patients and their partners to cope with these discrepancies and to estimate what information couples need. It is therefore pivotal to involve partners of patients in ACP and in shared decision-making regarding medical care and treatment options [41]. Our interview study included two single patients as well, who pointed out specific difficulties of living with the psychosocial consequences of their disease by themselves, without an intimate partner supporting them in everyday life. Increased awareness of these potential struggles for single patients could help HCPs to provide support.

In line with results from a recent study, we found that a prominent unmet psychosocial need of patients and partners was the lack of a (hospital based) caregiver or case manager to reach out to with physical and psychosocial issues [24, 42]. Patients sometimes felt lost in where to turn to, increasing distress. In some hospitals, CNSs, or so-called case managers, have a central role within the advanced cancer disease trajectory and function as key contact person for patients to reach out to with physical or psychosocial issues. Patients described their positive experiences, and the HCPs in our interview study elaborated on CNSs being suitable to function as key contact person for patients. However, currently the organisation of psychosocial oncological care in the Netherlands differs per hospital, and without a central contact person, the oncologist or GP remains the first HCP to reach out to in case of issues, which might lead to the patient feeling unheard because of the lack of up-to-date medical information or time for a consult at that time. In rheumatological care, nurses fill in an important central role [43]. They help patients and their loved ones to cope with their condition, provide psychosocial support to both, communicate with other involved disciplines, and they are perceived effective, safe and accessible by patients [44]. There are more examples of advanced nurse practitioners (ANP) in various health care domains, including in oncology [45]. ANP can decrease stress and fear and improve quality of life of cancer patients. In advanced cancer, a central role could be of added value to both patients and other HCPs involved in long-term care for patients. Further research should concentrate on how advanced nursing practice in advanced cancer could be organised where long-term guidance of patients is required, following examples in other health care domains [44, 45].

In a recent study, patients with increased levels of distress, depression and anxiety and lower levels of quality of life or social support reported higher unmet psychosocial needs [24]. In our interview study, patients experienced periods of distress, depression, fears, and lack of social support, but not all of them were in need for psychological help, and they perceived psychosocial issues to be normal given their circumstances. However, if psychosocial help was desired, patients did not always know where and how to arrange it. With an expected growing number of patients living longer with advanced cancer in the future, and the sometimes rapid changes in physical and psychosocial wellbeing, monitoring patients to timely identify when support is needed. Currently, no feasible instruments for advanced cancer are available that accurately assess the unique psychosocial challenges of living with advanced cancer and systemic treatment [21, 24, 27, 46]. Development of clinical instruments focusing on fear of progression and intolerance of uncertainty could help identify those in need for help. These instruments could be used by oncologists or CNSs to monitor patients during a longer-term disease trajectory, aimed at timely providing tailored psychosocial support.

### Strengths and limitations

The conduction of this interview study and reporting of the results aimed to be credible, transferable, dependable and confirmable [47]. The interviews were conducted by researchers with an open-minded attitude, recognising their personal biases and there was self-reflection throughout the entire study process facilitated by the research team (credibility). Furthermore, the researchers provided detailed and comprehensive explanations allowing for evaluation of applicability of finding to similar situations (transferability). Dependability was assured by log documentation of decisions made throughout the research process. Confirmability was ascertained by peer debriefing with colleagues within the research team with different professional backgrounds and expertise with this patient group to authenticate interpretations and mitigation of researcher bias. To maintain rigour in our research, we adhered to the COREQ guidelines [28]. A strength of this interview study was that psychosocial aspects and organisation of psychosocial care were broadly explored from different perspectives including patients, their partners, and various HCPs. By purposive sampling, we were able to recruit a diverse sample, including patients with varying advanced cancer diagnoses and treatments, and HCPs with varying backgrounds, working in primary care and the hospital. A limitation of purposive sampling via the patients’ treating HCP is that participant inclusion may be biased by HCPs’ assessment of the patients’ physical and/or psychosocial eligibility to perform well in an interview study, leading to a less representative sample. Patients and partners who were invited to participate were eager to contribute to improving psychosocial care for (future) fellow patients in advanced oncology, and they valued their enrolment in an interview study as an opportunity to share their personal story. Although patients’ willingness to contribute facilitated recruitment, there is a risk of selection bias. Participants willing to share their story might be more susceptible to enrol, but might differ in personal characteristics or psychosocial experiences in comparison to those not willing to participate in a psychosocial interview study. The participant sample was not diverse with regard to ethnicity and culture, as we were only able to include white, native Dutch participants. Information on culture or religious beliefs was not gathered. People with other cultures might have different psychosocial experiences, preferences and needs regarding treatment, care, and the end-of-life phase [48]. In addition, the experiences regarding the organisation of psychosocial care were discussed in the context of the health care setting in the Netherlands, which might limit generalisability of the results. In future research, experiences of patients, partners and HCPs with different cultural and religious backgrounds should be explored, and in the context of different health care settings.

The interviews took place during the COVID-19 pandemic in 2020, when restrictions made in-person interviews impossible at the start. Due to the vulnerability of the patient population and the unknown impact of COVID-19, prior to vaccination, and with a hospital policy to continue digital care when possible, we only conducted digital interviews. Differences between outcomes from video calls and face-to-face interviews are expected to be minimal [49, 50], and no major differences were found across outcomes in recent literature on psychosocial aspects of living with advanced cancer performed before the COVID-19 pandemic [19, 21, 51]. Yet, in a digital setting, it was complex for the researcher to control whether patient and partner were both present during the individually addressed interview questions, and although some couples preferred to be interviewed simultaneously, this could have prevented them to speak freely and with more comfort with their partner being present. However, in the last two interviews, patients and partners were interviewed separately and this did not lead to new themes.

### Conclusion and clinical implications

Insight in the unique psychosocial aspects of living longer with advanced cancer and systemic treatment from the perspectives of both patients and their partners is timely, since the number of people living with advanced cancer and systemic treatment is increasing. Patients experienced increased losses in several life domains, were confronted with complex life decisions, and lived with ongoing uncertainty and fluctuating levels of fear and hope. The reported challenges in organising optimal psychosocial support for patients with advanced cancer and their partners suggested improvements in the following domains:

- Psychosocial wellbeing, needs, goals and preferences regarding treatment, care and the end-of-life phase of both patient and partner should be addressed from an early stage on, engaging involved HCPs from both primary care and the hospital.
- A central hospital-based contact person for patients to reach out to with psychosocial (or physical) issues, for example a CNS or case-manager, could guide patients and partners over longer periods of time, thereby improving their psychosocial wellbeing and quality of life.
- The waiting time between undergoing a scan and the patient’s consultation with a specialist about the results should be limited as much as possible, aiming at decreasing scanxiety, fear and uncertainty.
- Instruments aimed at measuring psychosocial wellbeing of advanced cancer patients could help to identify those in need for psychosocial help.
- Awareness of and attention to the psychosocial wellbeing and unique struggles of partners of advanced cancer patients is pivotal, and they should be involved in ACP.

## Supplementary Information

Below is the link to the electronic supplementary material.

Supplementary file1 (PDF 415 KB)

Supplementary file2 (PDF 84 KB)

Supplementary file3 (PDF 123 KB)

Supplementary file4 (PDF 37 KB)

## Data Availability

The data that support the findings of this study are available from the corresponding author JC upon reasonable request.
