# Supplementary material for: Perspectives of patients, partners, primary and hospital-based health care professionals on living with advanced cancer and systemic treatment
Source: J Cancer Surviv. 2024 Oct 29;20(3):940–53. doi: 10.1007/s11764-024-01698-w (PMC13144172; doi:10.1007/s11764-024-01698-w)
Supplement: Supplementary file 2 — Supplementary file2 (PDF 84 KB) [file 11764_2024_1698_MOESM2_ESM.pdf]

Semi structured interview guide
# - Health care professionals (HCP) -

<u>TOPIC 1. Person, role, experience with patient group (Theme: personal)</u>

1.1 Personal situation
- Could you briefly introduce yourself?
- What is your role?
- Where are you employed?
- How long have you been in this role?
- Which patients do you treat? With which diagnoses?
- What is your experience in providing care for patients with advanced cancer requiring ongoing systemic treatment and monitoring?

<u>TOPIC 2. Psychosocial care (Theme: psychosocial care)</u>

2.1 Medical care
- What does the care pathway look like for advanced cancer patients requiring ongoing systemic treatment?
- What is your role in the care pathway?
- How does this role relate to that of the physician? *(in case of a cancer nurse specialist*)
- How often do you see these patients?
- What do patients contact you about? How frequently? What topics are discussed?
- How do you involve the patient's relatives in the care you provide?
- How do patients perceive the current care in your department (treatments, monitoring, scans)?

2.2 Scans/checks
- How often do scans/checks take place?
- What effect does the (frequency of) scans/checks have on the patient? What does it mean for the patient?

2.3 Psychosocial aspects
- Do you discuss the psychosocial consequences (emotions or thoughts/impact on daily life) that your patient experiences or may experience? Why or why not?
- What psychosocial aspects (emotions and thoughts) are present in these patients?
- Could you describe a patient?
- How do you approach this topic? What is important to you in this regard? Which topics do you consider important?
- Do you discuss this in general, or are there specific topics that you explore further? Why these topics?
- Is there anything that you find difficult to discuss? What and why?
- Is there enough time during the consultation to discuss everything?

- Do you feel that patients need to discuss the psychosocial consequences with their specialist? Or with another healthcare provider? (*in case of a nurse specialist*)

2.4 Referral process

- Do you refer patients for psychological support? Where to? How does the referral process work?
- Do you discuss the possibility of referral for psychological support with your patients? Why or why not?
- Do you notice differences in the occurrence or expression of psychosocial aspects among different patient groups/different diagnoses? For example, in relation to curative patients?

For psychosocial HCPs: TOPIC 2.2 Psychosocial care (Theme: psychosocial care)

- *What does the psychosocial care pathway for patients typically look like?*
- *How often do you see these patients?*
- *What is your role in the process?*
- *How do you involve the patient's family members in the care you provide?*
- *Through which route(s) do patients come to you (referral process)?*
- *What does the psychological support/treatment for these patients involve?*
- *What do you consider important in conversations with these patients?*
- *How do you involve the specialist, clinical nurse specialist, and/or GP or other HCPs in this?*
- *In your opinion, are psychosocial aspects/consequences addressed by the specialist?*
- *Do patients have the need to discuss the psychosocial consequences with their specialist?*

TOPIC 3. Psychosocial aspects in patients (Theme: thoughts, emotions, behaviour patient)

3.1 Thoughts, emotions, triggers, coping

- What is typical of the thoughts and/or emotions of patients with a form of cancer requiring continuous treatment and monitoring? How do these patients present themselves?
- What psychosocial aspects/consequences do these patients experience (anxiety, depression, uncertainty, hope)? Could you name (the most important) three?
- In what ways do patients express these feelings?
- What thoughts are associated with or underpin these emotions?
- How do patients cope with these thoughts and emotions?
- Can you identify any triggers that provoke these thoughts and emotions?
- Do you recognise existential or meaning-of-life questions in your patients?
- How do patients perceive the current (medical) care? (e.g. check-ups, scans)?
- What impact does the (frequency of) check-ups/scans have on the patient? What does this mean for the patient?
- Do you see any differences in the occurrence or expression of psychosocial aspects between other patient groups or across different diagnoses? For example, compared to curative patients?

3.2 Fear of progression

- Do you recognise fear of progression in these patients (as a primary concern)?
- What does that look like?
- How do you address it?
- Do you adjust the diagnosis/treatment based on it?

- Could you describe how a patient with severe fear of progression presents? What is characteristic of this patient? How does it manifest?
- What is their care need?
- How do patients cope with (severe) fear of progression?
- Can you name triggers for the fear of progression?
- Do you see any differences in the occurrence or expression of fear of progression compared to other patient groups/ different diagnoses? Or in comparison to curative patients?

TOPIC 4. Personal experiences (Theme: personal experiences)

- How do you experience the changing care pathway, in which you to treat some patients for a long period of time? How does that affect you?
- How do you manage this as a healthcare provider?
- Are there aspects of care or the relationship/communication between the specialist and this specific patient group that you would like to see change?
- Are there topics that you find difficult or challenging to discuss?
- What topics do you consider important to discuss?
- Is there anything you would advise other HCPs?
- Is there anything you would advise patients?
