# Supplementary material for: Perspectives of patients, partners, primary and hospital-based health care professionals on living with advanced cancer and systemic treatment
Source: J Cancer Surviv. 2024 Oct 29;20(3):940–53. doi: 10.1007/s11764-024-01698-w (PMC13144172; doi:10.1007/s11764-024-01698-w)
Supplement: Supplementary file 3 — Supplementary file3 (PDF 123 KB) [file 11764_2024_1698_MOESM3_ESM.pdf]

Semi structured interview guide
# - Partners -

<u>TOPIC 1. Personal (Theme: personal)</u>

1.1 Personal situation
- Could you tell me a bit more about yourself?
- How old are you?
- Where are you from?
- What is your family situation like?
- Do you work?/What have you done in the past?
- What does a typical day look like for you?

1.2 Diagnosis and treatment history partner
- What diagnosis has your partner received?
- When was this diagnosis made?
- What led up to this diagnosis?
- What did you think the diagnosis meant for your future? Was the prognosis discussed at the time?
- Were you present when your partner was diagnosed? Could you tell me a bit more about that?
- Where is your partner currently receiving treatment?
- What does the treatment involve/what has the treatment process been like?
- Which/how many doctors have been involved in the treatment?
- What effect has the treatment had on your partner? What have you noticed (are there any side effects)?
- What do you expect from the treatment?
- Has the treatment changed your expectations about the course of the illness?
- How often are there check-ups/appointments and what do they involve (with whom, duration, scans)?
- What do you expect from the check-ups?
- What impact do the check-ups have on you and your partner?
- How long does it usually take for your partner to receive the results, and how does he/she hear about them? How do you experience that?
- Would you prefer to have more or fewer check-ups?
- Are you involved in the treatment decisions your partner has to make? How do you feel about that?
- Is there anything you would like to see done differently in the treatment process?

1.3 Doctor-patient relation/communication
- Could you tell me a bit more about the contact you have with the (lead) treating physician(s)?
- What do you expect from the (lead) treating physician(s)?
- Would you prefer to speak to the doctor more or less often?
- What do you consider important in your contact with healthcare providers?
- Is there anything you would like to be different in your contact with healthcare providers?

1.4 Altered circumstances

- What are you dealing with now that your partner is ill?
- What has changed?
- How do you experience this/how is this for you?

<u>TOPIC 2. Psychological aspects (Theme: thoughts, emotions, behaviour)</u>

2.1 Thoughts, emotions, triggers, coping, behaviour

- Could you tell me a bit more about the thoughts or images you have regarding your partner's illness? What kind of thoughts or images are they? (the course of the illness, memories, treatment, death, or something else?)
- How often do these thoughts or images arise?
- When do these thoughts or images arise? What triggers them?
- What impact do these thoughts or images have on your daily life?
- What do you do when these thoughts or images come up? What are the consequences?
- What do you do to manage or control these thoughts or images?
- Do you feel that you have any influence over your partner's illness or its course? Is there a sense of control?
- What emotions/feelings are involved? (Examples include fear, hope, uncertainty, tension, relief, etc.)
- How often do these feelings arise?
- What impact do these feelings have on your daily life?
- What do you do when these emotions/feelings arise? What are the consequences?
- What do you do to manage or control these emotions/feelings?
- Are there factors that worsen these emotions/feelings?
- Do you avoid certain things because of this?
- Are there factors that make these thoughts/emotions/feelings disappear?

If no thoughts or images about a possible worsening of the illness have been mentioned, the presence of avoidance or denial must be explored:

- Do you do anything to prevent thoughts or images about your illness from arising?
- What would you advise other people with cancer who are struggling with distressing thoughts and images about the illness?

2.2 Fear of progression

- Are you ever afraid or worried that your partner's cancer will become active or grow?
- What are those thoughts like? How do those thoughts or worries manifest?
  How often does that happen?
- When do these worries arise? What triggers them?
- What impact does this fear have on your daily life?
- What emotions or feelings do you have with this?
- What do you do when these thoughts/feelings arise? What are the consequences?
- What do you do to control them?
- Are there factors that trigger or worsen the fear of the cancer returning or worsening?

- Do you avoid certain things because of this?
- Are there factors that make the fear disappear?

Also, further explore uncertainty if necessary, in the same way.

2.3 Existential issues/sense of meaning
- How does it affect you now that your partner is expected to live longer with his/her current therapy?
- What is particularly on your mind at the moment? What is important in your life?
- Do you think about what your future will look like (dealing with the loss of...)?
- Who or what provided you with support in previous situations?
- Who would you like to have with you at this moment for support?

<u>TOPIC 3. Social aspects (Theme: social)</u>

3.1 Family life
- How does your partner cope with his or her illness? What do you notice about that?
- What has changed in your relationship with your partner compared to when your partner was not ill? (Role changes: for example, having to work more, a different role for the children…)
- Are there certain things you do not discuss with your partner? (Avoidance or sparing feelings)
- Are there things you do discuss with your partner?
- Do you have children? How do your children cope with your partner and his/her illness? What do you notice about that?
- What helps you in this situation?
- What does not help you in this situation?
- Does your partner/family/social circle have concerns about the progression of the illness? What do you notice about that?

3.2 Social relationships
- How do others in your environment cope with you and your partner? What do you notice about that?
- What do you think about that?
- What helps you in your interactions with others?
- What does not help you in your interactions with others?
- To what extent do you involve your social circle in your partner's illness/treatment process?

3.3 Work
- Are you still working (full-time)? How is that going?
- Since your partner's illness, have you had to make any changes related to your work?
- How have you experienced your employer's reaction?
- How have you experienced your colleagues' reactions?

3.4 Daily life
- In the previous section, you indicated what has changed in your daily life. What would you still like to do in your daily life, and are you able to do that? If not, why not?
