# Supplementary material for: Perspectives of patients, partners, primary and hospital-based health care professionals on living with advanced cancer and systemic treatment
Source: J Cancer Surviv. 2024 Oct 29;20(3):940–53. doi: 10.1007/s11764-024-01698-w (PMC13144172; doi:10.1007/s11764-024-01698-w)
Supplement: Supplementary file 4 — Supplementary file4 (PDF 37 KB) [file 11764_2024_1698_MOESM4_ESM.pdf]

Semi structured interview guide
# - Patients -

TOPIC 1. Person, diagnosis, treatment pathway (Theme: personal)

1.1 Personal situation
- Could you tell me a bit more about yourself?
- How old are you?
- Where are you from?
- What is your family situation?
- Do you work/what have you done in the past?
- What does your day typically look like?

1.2 Diagnosis and treatment pathway
- What diagnosis were you given?
- When was this diagnosis made?
- What events led up to this diagnosis?
- What did you think the diagnosis meant for your future? Was the prognosis discussed at that time?
- Was there someone with you when you received the diagnosis?
- Where are you currently receiving treatment?
- What does the treatment look like, or what has the treatment process been like so far?
- How many doctors are, or have been, involved in your treatment?
- What effects does the treatment have on you? What do you notice? (Are there any side effects?)
- What do you expect from the treatment?
- Has the treatment changed your expectations regarding the course of your illness?
- How often do you have follow-up appointments, and what do these appointments involve (with whom, duration, scans)?
- What do you expect from these follow-up appointments?
- What impact do the follow-up appointments have on you?
- Does someone accompany you to your follow-up appointments?
- How long does it typically take before you receive results, and how are you informed of them?
- How do you experience this process?
- Would you prefer to have more or fewer follow-up appointments?
- Do you involve your family in treatment decisions?
- Is there anything you would like to see done differently in your treatment pathway?

1.3 Doctor-patient relationship/communication
- Could you tell me a bit more about the contact you have with your primary doctor(s)?
- What do you expect from your primary doctor(s)?
- Would you prefer to speak with your doctor(s) more or less frequently?
- What do you consider important in your communication with healthcare providers?

- Is there anything you would like to see done differently in your interactions with your healthcare providers?

1.4 Changed circumstances
- What has changed for you since you became ill?
- How do you experience these changes/how is this for you?

TOPIC 2. Psychological aspects (Theme: thoughts, emotions, and behaviour)

2.1 Thoughts, emotions, triggering factors, coping
- Could you tell me more about the thoughts or images you have regarding your illness? What kind of thoughts or images are those? (course of illness, memories, treatment, dying, other?)
- How often do these thoughts or images arise?
- When do these thoughts or images arise? What triggers them?
- What impact do these thoughts or images have on your daily life?
- What do you do when these thoughts or images come up? What are the consequences?
- What do you do to exercise control over these thoughts or images?
- Do you feel like you have any influence over your illness or its course? Do you feel a sense of control?
- What emotions/feelings arise in connection with your illness? (Examples could include fear, hope, uncertainty, tension, relief, etc.)
- How often do these emotions/feelings come up?
- What impact do these emotions have on your daily life?
- What do you do when these emotions/feelings arise? What are the consequences?
- What do you do to exercise control over these emotions/feelings?
- Are there factors that worsen these emotions/feelings?
- Do you avoid certain things because of these feelings?
- Are there factors that help these thoughts/emotions/feelings disappear?

If no thoughts or images regarding a potential worsening of the illness have been mentioned, the presence of avoidance or denial should be explored.
- Do you do anything to prevent thoughts or images about your illness from coming up?
- What advice would you give to other people with cancer who struggle with distressing thoughts and images about their illness?

2.2 Fear of progression
- Are you ever afraid or worried that the cancer may become active or grow?
- What kind of thoughts do you have? How do these thoughts or concerns manifest?
- How often does this occur?
- When do these worries or thoughts arise? What are the triggers?
- What impact does this fear have on your daily life?
- What emotions or feelings accompany this fear?
- What do you do when these thoughts arise? What are the consequences?
- What strategies do you employ to exert control over this fear?
- Are there factors that provoke or exacerbate your fear of recurrence or progression of the illness?

- Do you avoid certain things as a result?
- Are there factors that help alleviate this fear?

Additionally, continue probing into uncertainty if necessary, in the same manner.

2.3 Existential questions/meaning-making
- What impact has living longer with your current treatment had on you?
- What particularly occupies your mind at this moment? What is important to you in your life?
- Do you think about what your future will look like (coping with loss of...)?
- Do you reflect on the end of life or death?
- From whom or what did you draw support in previous situations?
- Who would you like to have with you at this moment for support?

<u>TOPIC 3. Social aspects (Theme: Social)</u>

3.1 Family situation
- How do you (as a single person), cope with your illness?
- What has changed in your relationship with your family? (Role changes: for example, needing to work more, a different role for the children...)
- Do you have children? How are your children coping with your illness? What do you notice about their reactions?
- What helps you in this situation?
- What does not help you?
- Does your family or social circle experience fear regarding the progression of your illness? What do you notice about this?

3.2 Social relationships
- How do others in your environment cope with your illness? What do you notice about their reactions?
- What are your thoughts on this?
- What helps you in your interactions with others?
- What does not help you in your interactions with others?
- To what extent do you involve your social environment in your illness or treatment process?

3.3 Work
- Are you still working? How is that going?
- How have you experienced your employer's response?
- How have you experienced your colleagues' reactions?
- If you are no longer working, how does that feel for you?

3.4 Daily life
- In the previous section, you mentioned what has changed in your daily life. What would you still like to do in your daily life, and are you able to do that? If not, what are the reasons?
